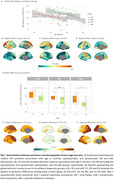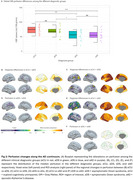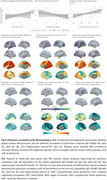# Study of brain perfusion in adults with Down Syndrome along the Alzheimer's Disease continuum

**DOI:** 10.1002/alz70856_106416

**Published:** 2026-01-09

**Authors:** Maria Franquesa‐Mullerat, Alejandra O. Morcillo‐Nieto, José Enrique Arriola‐Infante, Sara E Zsadanyi, Lídia Vaqué‐Alcázar, Mateus Rozalem Aranha, Javier Arranz, Íñigo Rodríguez‐Baz, Lucía Maure‐Blesa, Laura Videla, Isabel Barroeta, Laura Del Hoyo, Bessy Benejam, Susana Fernandez, Aida Sanjuan Hernandez, Sandra Giménez, Daniel Alcolea, Alberto Lleó, Maria Carmona‐Iragui, Juan Fortea, Alexandre Bejanin

**Affiliations:** ^1^ Sant Pau Memory Unit, Hospital de la Santa Creu i Sant Pau, Institut de Recerca Sant Pau ‐ Universitat Autònoma de Barcelona, Barcelona, Spain; ^2^ Center of Biomedical Investigation Network for Neurodegenerative Diseases (CIBERNED), Madrid, Spain; ^3^ Department of Medicine, Faculty of Medicine and Health Sciences, Institute of Neurosciences, University of Barcelona, Barcelona, Spain. Institut d’Investigacions Biomèdiques August Pi i Sunyer (IDIBAPS), Barcelona, Spain; ^4^ Neuroradiology Section, Department of Radiology, Hospital de la Santa Creu i Sant Pau, Biomedical Research Institute Sant Pau, Universitat Autònoma de Barcelona, Spain, Barcelona, Spain; ^5^ Barcelona Down Medical Center, Fundació Catalana Síndrome de Down, Barcelona, Spain; ^6^ Multidisciplinary Sleep Unit, Hospital de la Santa Creu i Sant Pau, Barcelona, Spain

## Abstract

**Background:**

Down Syndrome (DS) represents a high‐risk group for Alzheimer's disease (AD) due to chromosome 21 triplication, which drives amyloid precursor protein overproduction. While brain atrophy in DS has been widely studied, the underlying brain perfusion changes remain poorly understood. This study leverages MRI pseudo‐continuous arterial spin labeling (pCASL) to explore early cerebral blood flow (CBF) alterations along the AD continuum in DS and compares these changes to the perfusion patterns seen in sporadic AD (sAD)

**Method:**

We performed a cross‐sectional analysis including 32 euploid cognitively unimpaired individuals (eCU, age= 56.8yo, 68.7% female), 37 adults with DS (age= 42.64y; females= 37.83%, 40.5% symptomatic including *n* = 8 prodromal AD and *n* =  7 dementia) and 24 sAD patients (age 74.2yo, 50% female, 16 MCI and 8 in dementia stage) from the SPIN and DABNI cohorts that underwent 3T‐MRI. pCASL images were preprocessed using ASLprep. Analyses explored the effects of demographic variables (age, sex), clinical stages, and AD biomarkers (including cerebrospinal fluid [CSF] Aβ1‐42/Aβ1‐40 ratio, pTau‐181, and hippocampal volume) on global and regional CBF.

**Result:**

Age‐related decreases in cerebral blood flow (CBF) were observed in prefrontal regions in eCU, parietal structures in DS, and temporal lobes in sAD (Figure 1 A‐D). Females had higher perfusion than males in eCU and sAD, but not in DS (Figure 1 E‐H). In DS, CBF was reduced in temporal‐parietal regions in asymptomatic individuals, extending to frontal areas in symptomatic cases, resembling sAD patterns (Figure 2). Symptomatic DS showed significantly lower CBF in lateral parietal regions than asymptomatic DS. Temporoparietal CBF correlated negatively with CSF‐pTau‐181 and positively with CSF‐Aβ1‐42/Aβ1‐40 ratio and hippocampal volume, with the strongest association seen with hippocampal volume (Figure 3)

**Conclusion:**

Brain perfusion is significantly altered in adults with DS along the AD continuum, with changes detectable even before clinical symptoms. Hypoperfusion primarily affects temporoparietal and frontal regions at the symptomatic stage, closely resembling patterns seen in sAD. Notably, perfusion in the parietal areas differentiates asymptomatic from symptomatic DS and correlates strongly with key AD biomarkers. These findings highlight the potential of pCASL to detect early functional changes in this high‐risk population.